# Chitosan in Plant Protection

**DOI:** 10.3390/md8040968

**Published:** 2010-03-30

**Authors:** Abdelbasset El Hadrami, Lorne R. Adam, Ismail El Hadrami, Fouad Daayf

**Affiliations:** 1 University of Manitoba, Department of Plant Science, 222, Agriculture Building, Winnipeg, Manitoba, R3T 2N2, Canada; E-Mail: elhadram@cc.umanitoba.ca (A.E.); lorne_adam@umanitoba.ca (L.R.A.); 2 Laboratoire de Biotechnologies, Protection et Valorisation des Ressources Végétales (Biotec-VRV), Faculté des Sciences Semlalia, B.P. 2390, 40 000, Marrakech, Morocco; E-Mail: hadrami@ucam.ac.ma (I.E.)

**Keywords:** chitin, chitosan, biocidal activity, plant defenses, resistance, biological control

## Abstract

Chitin and chitosan are naturally-occurring compounds that have potential in agriculture with regard to controlling plant diseases. These molecules were shown to display toxicity and inhibit fungal growth and development. They were reported to be active against viruses, bacteria and other pests. Fragments from chitin and chitosan are known to have eliciting activities leading to a variety of defense responses in host plants in response to microbial infections, including the accumulation of phytoalexins, pathogen-related (PR) proteins and proteinase inhibitors, lignin synthesis, and callose formation. Based on these and other proprieties that help strengthen host plant defenses, interest has been growing in using them in agricultural systems to reduce the negative impact of diseases on yield and quality of crops. This review recapitulates the properties and uses of chitin, chitosan, and their derivatives, and will focus on their applications and mechanisms of action during plant-pathogen interactions.

## 1. Introduction

Both chitin and chitosan have demonstrated antiviral, antibacterial, and antifungal properties, and have been explored for many agricultural uses. They have been utilized to control disease or reduce their spread, to chelate nutrient and minerals, preventing pathogens from accessing them, or to enhance plant innate defenses.

When used to enhance plant defenses, chitin and chitosan induce host defense responses in both monocotyledons and dicotyledons. These responses include lignification [1], ion flux variations, cytoplasmic acidification, membrane depolarization and protein phosphorylation [2–5], chitinase and glucanase activation [6,7], phytoalexin biosynthesis [8,9], generation of reactive oxygen species [10], biosynthesis of jasmonic acid [11], and the expression of unique early responsive and defense-related genes [12–14]. In addition, chitosan was reported to induce callose formation [15,16], proteinase inhibitors [17], and phytoalexin biosynthesis [18] in many dicot species. The response to chitin, chitosan, and derived oligosaccharides varies with their acetylation degree. This review summarizes some of the uses of these natural products in agriculture and gives an overview of the mechanisms involved.

## 2. Antimicrobial Properties of Chitosan

Chitosan exhibits a variety of antimicrobial activities [19–21], which depend on the type of chitosan (native or modified), its degree of polymerization, the host, the chemical and/or nutrient composition of the substrates, and environmental conditions. In some studies, oligomeric chitosans (pentamers and heptamers) have been reported to exhibit a better antifungal activity than larger units [20]. In others, the antimicrobial activity increased with the increase in chitosan molecular weight [21], and seems to be faster on fungi and algae than on bacteria [22].

### 2.1. Against viruses

Chitosan was shown to inhibit the systemic propagation of viruses and viroids throughout the plant and to enhance the host’s hypersensitive response to infection (19,23,24]. The level of suppression of viral infections varied according to chitosan molecular weight [21]. Similar observations were reported with the potato virus X, tobacco mosaic and necrosis viruses, alfalfa mosaic virus, peanut stunt virus, and cucumber mosaic virus [19,24–27].

### 2.2. Against bacteria

Chitosan inhibits the growth of a wide range of bacteria [28]. The minimal growth-inhibiting concentrations vary among species from 10–1,000 ppm. Quaternary ammonium salts of chitosan, such as *N*,*N*,*N-*trimethylchitosan, *N-*propyl-*N*,*N-*dimethylchitosan and *N-*furfuryl-*N,N-*dimethylchitosan were shown to be effective in inhibiting the growth and development of *Escherichia coli* [29], especially in acidic media. Similarly, several derivatives of chitin and chitosan were shown to inhibit *E. coli, Staphylococcus aureus* [30], some *Bacillus* species, and several bacteria infecting fish.

### 2.3. Against fungi and oomycetes

Fungicidal activity of chitosan has been documented against various species of fungi and oomycetes [28,31]. The minimal growth-inhibiting concentrations varied between 10 and 5,000 ppm [32–36]. The maximum antifungal activity of chitosan is often observed around its *pKa* (pH 6.0).

Rabea *et al.* [37], reported on the fungicidal activity of 24 new derivatives of chitosan (*i.e.*, *N-*alkyl, *N*-benzylchitosans) and showed, using a radial hyphal growth bioassay of *B. cinerea* and *P. grisea*, that all derivatives have a higher fungicidal action than the native chitosan. *N*-dodecylchitosan, *N*-(*p*-isopropylbenzyl)chitosan and *N*-(2,6-dichlorobenzyl)chitosan were the most active against *B. cinerea*, with EC_50_ values of 0.57, 0.57 and 0.52 g.L^−1^, respectively. Against *P. grisea*, *N*-(*m*-nitrobenzyl)chitosan was the most active, with 77% inhibition at 5 g.L^−1^. *O*-(decanoyl)chitosan at mol ratio of 1:2 (chitosan to decanoic acid) was the most active compound against *B. cinerea* (EC_50_ = 1.02 g.L^−1^) and *O*-(hexanoyl)chitosan displayed the highest activity against *P. grisea* (EC_50_ = 1.11 g.L^−1^). Some of the derivatives also repressed spore formation at rather high concentrations (1.0, 2.0 and 5.0 g.L^−1^) [38]. Recently, Palma-Guerrero *et al.* [39] demonstrated that chitosan is able to permeabilize the plasma membrane of *Neurospora crassa* and kills the cells in an energy-dependent manner.

In general, chitosan, applied at a rate of 1 mg/mL, is able to reduce the *in vitro* growth of a number of fungi and oomycetes except Zygomycetes, which have chitosan as a component of their cell walls [40]. Another category of fungi that seems to be resilient to the antifungal effect of chitosan, the nemato-/entomo-pathogenic fungi that possess extracellular chitosanolytic activity [41].

### 2.4. Against insects

As more and more derivatives of chitosan (*i.e.*, *N-*alkyl-, *N*-benzylchitosans) are made available through chemical synthesis, their insecticidal activities are being reported using an oral larvae feeding bioassay [37,38]. Twenty four new derivatives were shown to have significant insecticidal activity when administered at a rate of 5 g·kg^−1^ in an artificial diet [37]. The most active derivative, *N*-(2-chloro-6-fluorobenzyl)chitosan, caused 100% mortality of larvae and its LC_50_ was estimated at 0.32 g.kg^−1^. All synthesized derivatives highly inhibited larvae growth as compared to chitosan by 7% and the most active derivative was the *O*-(decanoyl)chitosan, with 64% growth inhibition after 5 days of feeding on the treated artificial diet.

## 3. Applications of Chitosan in Plant Disease Control

Chitosan used to control plant pathogens has been extensively explored with more or less success depending on the pathosystem, the used derivatives, concentration, degree of deacylation, viscosity, and the applied formulation (*i.e.*, soil amendment, foliar application; chitosan alone or in association with other treatments). For example, Muzzarelli *et al.* [42] tested the effectiveness of five chemically-modified chitosan derivatives in restricting the growth of *Saprolegnia parasitica*. Results indicated that methylpyrrolidinonechitosan, *N*-phosphonomethylchitosan, and *N*-carboxymethylchitosan, as opposed to *N*-dicarboxymethylchitosan, did not allow the fungus to grow normally.

Substratum amendment with chitosan was reported to enhance plant growth and suppress some of the notorious soil-borne diseases. For example, in soilless tomato, root rot caused by *Fusarium oxysporum* f. sp. *radicis-lycopersici* was suppressed using chitosan amendments [43]. Similarly, in order to control post-harvest diseases, addition of chitosan stimulated microbial degradation of pathogens in a way resembling the application of a hyper-parasite [44]. This area of application is important because it suggests alternatives to the use of pesticides on fresh produce in storage [45,46]. Recent investigations on coating tomatoes with chitosan have shown that it delayed ripening by modifying the internal atmosphere, which reduced decays due to pathogens [45,46]. Various methods of application of chitosan and chitin are practiced to control or prevent the development of plant diseases or trigger plant innate defenses against pathogens.

### 3.1. Applied as seed coating agents

Guan *et al.* [47] examined the use of chitosan to prime maize seeds. Although chitosan had no significant effect on germination under low temperatures, it enhanced germination index, reduced the mean germination time, and increased shoot height, root length, and shoot and root dry weights in two tested maize lines. In both tested lines, chitosan induced a decline in malonyldialdehyde content, altered the relative permeability of the plasma membrane and increased the concentrations of soluble sugars and proline, and of peroxidase and catalase activities.

In other studies, seed priming with chitosan improved the vigor of maize seedlings [48]. It was also reported to increase wheat seed resistance to certain diseases and improve their quality and/or their ability to germinate [49]. Similarly, peanut seeds soaked in chitosan were reported to exhibit an increased rate of germination and energy, lipase activity, and gibberellic acid and indole acetic acid levels [50]. Ruan and Xue [51] showed that rice seed coating with chitosan may accelerate their germination and improve their tolerance to stress conditions. In carrot, seed coating helps restrain further development of Sclerotinia rot [52]. Chitosan has also been extensively utilized as a seed treatment to control *F. oxysporum* in many host species [20].

### 3.2. Applied as foliar treatment agents

Foliar application of chitosan has been reported in many systems and for several purposes. For instance, foliar application of a chitosan pentamer affected the net photosynthetic rate of soybean and maize one day after application [53]. This correlated with increases in stomatal conductance and transpiration rate. Chitosan foliar application did not have any effect on the intercellular CO_2_ concentration. The authors reported that the observed effect on the net photosynthetic rate is, in general, common in maize and soybean after foliar application of high molecular weight chitosan. Foliar applications of these oligomers did not, on the other hand, affect maize or soybean height, root length, leaf area, or total dry mass.

Bittelli *et al.* [54] suggested that chitosan might be an effective anti-transpiring to preserve water resources use in agriculture. In their investigation, they examined the potential of foliar applications of chitosan on pepper plants transpiration in the growth room and in the field. In both experiments, the authors monitored plant water use directly and indirectly. The plant biomass and yield were determined to calculate biomass-to-water ratios and the differences in canopy resistance between control and chitosan-treated plants were analyzed. Using scanning electron microscopy and histochemical analyses, stomata were shown to close in response to treatment with chitosan, resulting in a decrease in transpiration. Reduced water use of pepper plants upon treatment with chitosan was estimated at 26–43%, while there was no change in biomass production or yield [54].

Iriti *et al.* [55] unveiled some of the aspects through which chitosan was able to reduce transpiration in bean plants after being used as a foliar spray. The authors showed that this activity was likely occurring thanks to the increase in abscisic acid (ABA) content in the treated leaves. Using scanning electron microscopy and other histocytochemistry techniques, the authors showed that upon treatment and increase in ABA content, a partial stomatal closure occurred and led, among others, to a decrease in conductance for water vapor and in the over all transpiration rate. Interestingly, the authors revealed a new chitosan anti-transpirant mechanism in bean plants that was not described by their commercial supplier Vapor Gard^®^, and in which a formation of a thin anti-transpirant film at the surface of the leaves was much more efficient than stomatal closure. This difference in mechanisms also suggested an important consideration for the environmental conditions under which chitosan is applied as shown by the authors but may also depends on the intrinsic properties of the tested plant species.

Chitosan has also been extensively utilized as a foliar treatment to control the growth, spread and development of many diseases involving viruses, bacteria, fungi and pests [20]. It has also been used to increase yield and tuber quality of micropropagated greenhouse-grown potatoes [56]. Similarly, Faoro *et al.* [57] showed that the use of chitosan applied as a foliar spray on barley reduced locally and systemically the infection by powdery mildew pathogen *Blumeria graminis* f. sp. *hordei*.

### 3.3. Applied as soil amendment

Chitosan utilized as a soil amendment was shown to control *Fusarium* wilts in many plant species [20]. Applied at an optimal concentration, this biomaterial is able to induce a delay in disease development, leading to a reduced plant wilting [58]. Similar results were reported in forest nurseries suffering from *F. acuminatum* and *Cylindrocladium floridanum* infections. These infections were dramatically reduced upon the use of chitosan as soil amendment [59]. *Aspergillus flavus* was also completely inhibited in field-grown corn and peanut after soil treatment with chitosan [45]. Part of the effect observed by chitosan on the reduction of soilborne pathogens comes from the fact that it enhances plant defense responses. The other part is linked to the fact that this biopolymer is composed of polysaccharides that stimulate the activity of beneficial microorganisms in the soil such as *Bacillus,* fluorescent *Pseudomonas*, actinomycetes, mycorrhiza and rhizobacteria [60,61]. This alters the microbial equilibrium in the rhizosphere disadvantaging plant pathogens. Beneficial organisms, on the other hand, are able to outcompete them through mechanisms such as parasitism, antibiosis, and induced resistance [62–65].

Vruggink [66] reported on the effect of chitin amendment on actinomycetes in soil and on the infection of potato from susceptible cultivar ‘Bentje’ by *Streptomyces scabies*, the causal agent of tuber scab. The percentage scab on tubers from the control and the soil amended with antagonist was about 22 % while only 4% of the tubers from the soil amended with chitin and chitin with antagonist had scab at harvest. After planting these tubers, for a second time, the scab was 21% on tubers from untreated soil and 9.5 % from soil amended with chitin. Investigation of the effect of chitin amendment on the actinomycete population in the soil, a few months after chitin amendment, revealed that chitin had a greater increase in total actinomycete population (24–30 times as compared to the untreated control). The study also showed that some actinomycetes (*i.e.*, *Micromonospora*) had disappeared, while others including *S. scabies* were isolated less frequently.

## 4. Mechanisms of Action of Chitosan in Reducing Plant Diseases

Although the exact mechanisms of action of chitosan in reducing plant disease are currently not fully understood, there is growing evidence showing its action through direct toxicity or chelation of nutrients and minerals from pathogens. Because of its biopolymer properties, this compound can also form physical barriers around the penetration sites of pathogens, preventing them from spreading to healthy tissues. This and bioactive derivatives can activate H^+^-ATPases, depolarizing biological membranes and inducing other series of events. Chitosan is known to induce reactions locally and systemically that involve signaling cascades, and the activation and accumulation of defenses-related antimicrobial compounds and proteins.

### 4.1. Direct activity against pathogens

Direct activity of chitosan against viruses and viroids has been shown to vary according to molecular weight [21]. However, none of the studies that investigated this effect has clearly proven the ability of chitosan in completely inactivating viruses or viroids. Most literature *i.e.*, [21] reported on the inactivation of replication, which lead to the stoppage of multiplication and spread. This could be linked to the fact that upon penetration into plant tissues, chitosan nanoparticles tightly bind nucleic acids and cause a variety of damages and selective inhibitions. For instance, the selectively exerted inhibition could inactivate the synthesis of essential mRNA encoded by various genes required for important metabolic and infectious processes of the virus or viroid. These properties have been largely explored in gene therapy and gene silencing [20,67].

Against, bacteria, fungi, oomycetes and other pests, it seems that chitosan is likely to operate indirectly *via* other means such as the enhancement of host resistance. However, a number of studies have shown that chitosan, at defined concentrations, presents antimicrobial properties [33,68,69]. For instance, chitosan was reported to exert an inhibitory action on the hyphal growth of numerous pathogenic fungi, including root and necrotrophic pathogens, such as *Fusarium oxysporum*, *Botrytis cinerea*, *Monilina laxa*, *Alternaria alternata* and *Pythium aphanidermatum* [63,70–76] besides inhibiting spore germination in some of them [18].

Chitosan is often used in plant disease control as a powerful elicitor rather than a direct antimicrobial or toxic agent. Its direct toxicity remains dependent on properties such as the concentration applied, the molecular weight, degree of acetylation, solvent, pH and viscosity [77,78]. The degree of acetylation defines the sites with which nucleophilic groups could react and viscosity provides an environment that could extend the duration and intensity of reactions.

### 4.2. Physical barrier around pathogen penetration sites

Chitosan, when applied to plant tissues, often agglutinate around the penetration sites and has two major effects. The first one is the isolation of the penetration site through the formation of a physical barrier preventing the pathogen from spreading and invading other healthy tissues. This phenomenon resembles the abscission zones often observed on leaves preventing several necrotrophic pathogens from spreading further. It is widely observed on potato tubers for example [79]. Around the isolated zones, often an elicitation of a hypersensitive response occur with the accumulation of H_2_O_2_ that helps in cells wall fortification and serve as an alert signal for other healthy parts of the plant. The second effect is due to the chitosan’ ability to bind various materials and initiate fast the wound healing process [80].

### 4.3. Chelation of nutrients and minerals

Chitosans are well used in the fresh and salt water purification process as chelators for minerals and metals. These abilities are also explored when chitosan is applied to plants to prevent diseases because it can chelate nutrients and minerals (*i.e.*, Fe, Cu), preventing pathogens from accessing them. These polysaccharide molecules were also reported to bind mycotoxins [81], which may reduce damage to the host tissues due to toxins. In the beverage industry, for example, chitosan and derivatives are often used for their antimicrobial properties linked to their chelating abilities of nutrient and minerals, thus reducing fungal spoilage [35].

### 4.4. Effect on H^+^-ATPase and depolarization of biological membranes

Amborabé *et al.* [82] reported on the early events that occur during the elicitation of plant defenses using chitosan. They showed that this molecule was able to trigger, in a dose-dependent manner, a quick and transient depolarization of *Mimosa pudica* motor cell membranes. These modifications were also accompanied by a transient rise in pH. Using plasma membrane vesicles, the authors determined the site of action of this polysaccharide to be the plasma membrane H^+^-ATPase due to the inhibitory effect observed on the proton pumping and the catalytic activity of the enzyme. Chitosan was also shown to alter many other H^+^-mediated processes [82]. For example, the uptake of certain carbohydrate and amino-acids was altered because of their dependence on co-transporters involving an exchange with H^+^. Similarly, the light-induced H^+^/K^+^-mediated turgor reaction was shown to be inhibited in *M. pudica* motor cells in response to the treatment with chitosan.

Ultra-structural studies conducted by Benhamou [71] have shown that treatment with chitosan induces a series of morphological and structural modification, leading to disorganized hyphae associated with inhibition of fungal growth. This was linked to the polycationic properties of chitosan, allowing for changes in terms of membrane permeability and cytoplasmic aggregation. As a consequence, the activities of a number of enzymes involved in the synthesis and assembly of cell wall polymers are disturbed [83].

### 4.5. Modulation of plant responses and signaling

Chitosan and derivatives are known to act as potent inducers, enhancing a battery of plant responses both locally around the infection sites and systemically to alert healthy parts of the plant. These include early signaling events as well as the accumulation of defense-related metabolites and proteins such as phytoalexins and PR-proteins [49,79,84–86]. Modulation of plant responses using chitosan has been reported in many pathosystems involving various plant species and a diverse range of pathogens, including virus and viroids, bacteria, fungi, nematodes and other pests [20,63,73,87,88]. This biopolymer was shown to be an effective inducer of phytoalexins synthesis and accumulation in various host cells [18,89], and triggers callose formation [15,16,83], lignification responses [80], and the production of proteinase inhibitors [17,88].

El Hassni *et al.* [63] studied the effect of chitosan in date palm in response to *Fusarium oxysporum* f. sp. *albedinis*, the causal agent of a major wilt in this crop. Beside a direct toxicity of the molecule on the fungus, the authors showed an enhancement of essential components of the host resistance. When injected into the roots at various concentrations, chitosan elicited date palm peroxidase and polyphenoloxidase activities, and increased the level of phenolic compounds. Among the accumulated phenolics, there was an increase in content of specific non-constitutive hydroxycinnamic acid derivatives, known to be of great importance in the resistance of this plant to this vascular fusariosis. Similarly, treatment of wheat seeds with chitosan revealed an increase in hydroxycinnamic (*i.e.*, *p-*coumaric, caffeic and ferulic) and benzoic (*i.e.*, benzoic, protocatechuic and gallic) acid derivatives, leading to an increase in lignin synthesis and accumulation [49]. PAL activity was also reported to increase in response to elicitation with chitosan in many host species [74,90].

Ramonell *et al.* [91] used a microarray consisting of 2,375 EST clones representing putative defense-related and regulatory genes to characterize changes in the gene expression patterns of *A. thaliana* in response to treatment with chitin. The authors reported that 71 ESTs, representing 61 genes, were altered three-fold or more in their transcript levels in chitin-treated seedlings as compared to the control. Interestingly, the levels of transcription of numerous genes were revealed to be altered as early as 10 min after exposure to chitin, hence translating the earliest changes that may occur in chitin-treated plants. These genes included commonly elicited defense-related genes (*i.e.*, phenylalanine amonia-lyase, chitinase, peroxidase) as well as other genes with function not yet identified. Among the transcriptional regulators, the authors reported on the increase in transcript accumulation of elements at the promoters region rich in W-boxes along with other unknown regulatory elements. In parallel, Ramonell *et al.* [91] showed a decrease in transcript abundance of a number of genes encoding cell wall strengthening and wall deposit proteins. These genes were all downstream the chalcone synthase promoter, suggesting their potential suppression during plant x pathogen interactions. The authors also examined the genes based on their controlling pathways. They found that among the up-regulated genes in response to treatment with chitin, there were 43% that were also up-regulated with salicylic acid, 39% with methyl jasmonate and another 36% with ethylene. Among the down-regulated genes in response to chitin, 7% shared the down-regulation with salicylic acid, 9% with methyl jasmonate and 14% with ethylene.

Similarly, Akimoto-Tomiyama *et al.* [92] examined the expression of defense-related genes in rice treated with *N*-acetylchitooctaose, using microarray analysis consisting of 8,987 randomly selected expressed sequence tags. In their experiments, the authors reported on the significant up-regulation of 166 genes and down-regulation of 93 genes. Out of the 259 responsive ESTs to *N*-acetylchytooctaose identified, the authors highlighted 18 putative genes related to signal transduction, including five calcium-dependent protein kinases (CDPKs).

### 4.6. Chitosan–A general pathogen-associated molecular pattern

Plants possess mechanisms by which they recognize their intruders. They are thought to have trans-membrane pattern recognition receptors (PRRs) able to interact with pathogen/microbe-associated molecular patterns PAMPs/MAMPs [93]. PAMPs/MAMPs can be any effectors secreted by the pathogens or released from the cell wall of the host upon attack on the infection site. Cell wall polysaccharides such as glucans and chitosan have been reported to act as PAMPs/MAMPs in many pathosystems. Chitosan presents the advantage of being recognized by plant PRRs and triggers a panel of defense responses. Iriti and Faoro [94] reported that chitosan behaves like a PAMPs/MAMPs or a general elicitor, inducing non-host resistance and priming systemic immunity. The defense responses enhanced by chitosan application include the increase in H^+^ and Ca^2+^ influx into the cytosol, the activation of MAP-kinases, callose apposition, oxidative burst, hypersensitive responses, the synthesis of abscisic acid, jasmonates, phytoalexins, and PR-proteins [82].

It was long believed that the elicitor activity of chitosan is mediated through the interaction of this polycationic molecule with negatively-charged phospholipids, rather than a specific interaction with a receptor-like molecule [95]. However, Day *et al.* [96], examining the expression patterns of two GRAS family genes responsive to chitosan, have suggested that these two genes were regulated, at least partially, by high-affinity chitin-binding proteins localized in the plasma membrane of rice [97,98]. Recently, several chitosan-binding proteins have been isolated and described as putative receptors for chitosan. These proteins are thought to bind also to chitin and have been called chitin elicitor-binding protein (CEBiP) [99]. However, the biological activity of chitosan, as a general elicitor, remains tied to its physicochemical properties such as the molecular weight, deacetylation degree and viscosity. These properties can make the difference between cytotoxicity due to higher concentrations and the priming of resistance using appropriate molecular weight, deacetylation degree, viscosity and concentration.

### 4.7. Effect on nuclear distortion and cell death

Chitosan induces programmed-cell death (PCD) and hypersensitive-associated responses in plants [100]. It induced chromatin condensation and marginalization followed by a destruction of the nuclei and subsequent inter-nucleosomal DNA fragmentation. It did not affect stomatal guard cells but affected epidermal cells. Anaerobic conditions prevented the chitosan-induced destruction of epidermal cells’ nuclei. The antioxidants nitroblue tetrazolium or mannitol suppressed the effects of chitosan, H_2_O_2_, or chitosan + H_2_O_2_ on epidermal cells. Using a series of inhibitor assays, the same authors demonstrated that chitosan-induced epidermal cell death involves reactive oxygen species generated by the NADPH oxidase of the plasma membrane. For example, the alternative oxidase inhibitors propyl gallate and salicylhydroxamate prevented chitosan-induced destruction of epidermal cells nuclei; and the plasma membrane NADPH oxidase inhibitors diphenylene iodonium and quinacrine abolished chitosan-induced destruction of epidermal cells nuclei. The mitochondrial protein synthesis inhibitor lincomycin removed the destructive effect of chitosan on epidermal cells nuclei. Likewise, the use of autophagy inhibitor 3-methyladenine removed the chitosan effect as an inducer of epidermal cells death.

Zuppini *et al.* [101] studied, using soybeans (*Glycine max* L.) cell, the mechanism of programmed cell death mediated by calcium and triggered by chitosan. The authors showed that concentration as low as 50 μg per mL prompted a massive influx of calcium into the cytosol along with an up-regulation of the gene encoding for the chalcone synthase, a release of H_2_O_2_ into the culture media, and cell death. According to this study, the cell death phenomenon occurred through the activation of the PCD pathway since the authors observed a substantial reduction of the cytoplasm and a condensation of the chromatin as well as an increase in the activity of proteases (*i.e.*, caspase 3-like protease), especially when chitosan was applied at higher doses. Chelation of the extracellular calcium was also shown by the authors to prevent PCD and other associated events from occurring.

Similar results were reported by Choi *et al.* [102] and Iriti *et al.* [103]. Studying the antiviral activity induced by chitosan in tobacco, Iriti *et al.* [103] reported that treatments of tobacco plants with 0.1% chitosan reduced significantly the spread of the necrosis virus and induced callose deposits, micro-oxidative bursts and micro-hypersensitive responses. Staining techniques used revealed typical morphological features of apoptosis including cytoplasm shrinkage and nuclear chromatin condensation. Cell death kinetic induced by chitosan was also shown to be delayed by Verapamil^®^, a calcium channel blocker. Genomic DNA showed inter-nucleosomal fragmentation with a distinct DNA-laddering pattern.

Using *Arabidopsis* cell suspensions, Cabrera *et al.* [104] reported that the size, the degree of acetylation and the concentration of the applied chitooligosaccharide elicitors was a key determinant for the switching between the phenylpropanoid pathway relying on the activation of PAL and cell death involving the production of peroxides.

### 4.8. Activated oxygen species -scavenging and antioxidant activity

In recent years, a growing attention has been directed towards the antioxidant activity of chitosan [105–108]. Water-soluble chitosan was shown to be an excellent scavenger of hydroxyl radicals, H_2_O_2_ and anion superoxide. The 50% inhibition concentrations (IC_50_) values ranged from 246 to 498 mg/mL [105] and most of the activity was attributed to the chitosan contents of hydroxylated amino- and other substituting groups [107]. The scavenging rate increased with the applied concentrations of chitosan [105]. Sun *et al.* [106] tested the antioxidant activity of several chitosan oligomers with various molecular weights and determined the IC_50_ of their scavenging ability against superoxide anion and hydroxyl radicals. The lowest scavenging abilities against superoxide anion and hydroxyl radicals were recorded with the oligomer having the highest tested molecular weight. Meanwhile, chitosan oligomers with lower molecular weight exhibited a better antioxidant activity. Chen *et al.* [108] reported on the improvement of the antioxidant activity of chitosan for medical uses through grafting of essential oil component eugenol and carvacrol to the chitosan nanoparticles.

### 4.9. Wound-healing accelerators

Because of their ability to adhere to biological membranes and other biomolecules and their positive changes, chitosan and derivatives are excellent supports for healing wounds produced upon mechanical injuries or pathogen attacks. Application of dressings made of chitin derivatives on injured tree-barks resulted in a faster wound healing [80]. As a general elicitor, chitosan is also reported to activate the synthesis and accumulation of a series of PR-proteins and defense-related proteins among which phenylalanine ammonia-lyase and peroxidase. Given the involvement of these two enzymes in the synthesis and assembly of lignin matrix and in the formation of tylloses, chitosan seems to accelerate the process of wound healing.

### 4.10. Chitosan and the octadecanoic pathway

The octadecanoic pathway represents the series of metabolic steps through which jasmonates are synthesized following oxidation of linolenic acid. This pathway has long been proposed a part of the signaling cascade that mediates plant defense responses after elicitation with oligouronide and polypeptide signals, resulting from insect and pathogen invasions. Doares *et al.* [110] reported on the importance of this pathway in signaling induced by oligosaccharides. The authors showed that the accumulation of inducible proteinase inhibitors in tomato, upon leaf treatment with fungal-derived chitosan oligosaccharides, was significantly reduced, when salicylic or diethyldi-thiocarbamic acids (SA and DIECA) were applied. The latter compounds were suggested to interfere with the octadecanoic pathway. Application of chitosan to plants through cut stems, led to a rapid increase in jasmonic acid content, confirming the activation of the octadecanoic pathway.

Chitosan is also reported to increase the endogenous levels of 2-oxo-phytodeionic and jasmonic acids in many species including rice [111], leading to the activation of the octadecanoic acid pathway. The defense responses relying on this pathway include chitinase and glucanase activities that have been shown to be induced by chitosan in *Citrus* and *Fragaria* species [112,113], lipoxygenase [114,115] and the accumulation of phytoalexins [49,79,116].

### 4.11. Chitin as a stimulator of pathogens’ effectors

Recent developments in fungal effectors have raised several questions regarding the interaction that chitin may have with certain secreted proteins and effectors. Many described fungal effectors are cysteine-rich proteins that are often secreted and play a role in virulence (*i.e.*, Avr2 and Avr4 of *Cladosporium fulvum* [117]). These two proteins are inhibitors of plant cysteine proteases and help protect chitin and the integrity of fungal cell walls against plant chitinases. It is likely that application of chitosan within a plant protection program against these fungal species is to interfere with this process of recognition of the effectors and their cognate counterparts. de Jonge and Thomma [118] have reported on lysine motifs (LysMs), known in prokaryotes and plants as carbohydrate-binding protein modules, and their importance during many plant × pathogen interactions [119]. The authors demonstrated that the putatively secreted LysM-containing proteins were widespread among fungi. They also proposed a model according to which these putative LysM effectors would play a role in sequestering by-products of degradation of the chitosan from the fungal cell walls, hence triggering host immunity to dampen host defenses. Therefore, using chitosan as an effective enhancer of plant defense responses should be well planned taking into account all these interactions.

### 4.12. Physiology and degradation of chitosan by pathogens

Gooday [120] reported on the physiology and degradation of chitin and chitosan by microorganisms. Degradation of these oligosaccharides is mainly due to bacteria and fungi that exhibit either chitinases or chitosanases. Chitinases are chitinolytic enzymes that break chitin and are found in many fungi (*i.e.*, *Trichoderma*), while extracellular chitosanases hydrolyze chitosan and were found in many nemato-/entomo-pathogenic fungi [41].

The biochemical pathways of degradation of chitosan have been reviewed by Davis and Eveleigh [121]. Organisms that degrade chitin exhibit exo- or endo-chitinase activities that hydrolyze glycosidic bonds in reactions referred to as chitinolytic. Deacytelation of chitosan, on the other hand, through the action of chitosanases appear to be important in environments where chitosan is a major component *i.e.*, estuarine sediments [122].

A number of soilborne fungi have been reported to exhibit a chitinolytic activity that surpasses that of bacteria. The most common ones belong to the group of Mucorales, especially *Mortierella* spp., Deuteromycetes and Ascomycetes, especially the genera *Aspergillus, Trichoderma, Verticillium, Thielavia, Penicillium* and *Humicola* [120]. In some of these species, chitinolytic activity is triggered upon sensing chitin-containing material [123].

## 5. Concluding Remarks

In an era of high demand for blemish-free food and high cost inputs, sustainable agriculture has only a slim margin to make profits while guaranteeing food supply to a growing population. The recourse to naturally-occurring products with interesting antimicrobial and eliciting properties such as chitin and chitosan and their derivatives has been getting more attention in recent years. These products can be used in a numbers of ways to reduce disease levels and prevent the development and spread of pathogen, thus preserving yield and quality. Interesting theoretical and applied findings were gathered in recent years and more are needed to examine the mechanisms governing the mode of action of these compounds pathosystem by pathosystem, when applied at large scales. Examination of better ways to incorporate these natural products into Integrated Pest Management strategies remains to be pursued in many major crops (*i.e.*, potatoes, canola) especially against soilborne diseases. Progresses in recent years allowed also for some understanding of the interactions between the chitosan effect and the octadecanoic pathway as well as the identification of the so-called chitin elicitor-binding proteins. These will lead to design specific chitin/chitosan applications/formulations suitable for various stages of plant growth and development in order to achieve a better control of a specific disease or a complex of co-habitant diseases (*i.e.*, potato early dying complex). From a co-evolutionary point of view, the extensive genomic and proteomic data gathered in many pathosystems, highlighting the secretion of fungal effectors able to inhibit plant cysteine proteases and protect chitin and cell wall integrity against plant chitinases, suggest that it will be of interest to examine how application of chitin and chitosan derivatives could interfere with the recognition of these effectors by their cognate counterparts.
